# A patient presenting with concha bullosa in another concha bullosa: a case report

**DOI:** 10.1186/1752-1947-6-87

**Published:** 2012-03-26

**Authors:** Ibrahim Cukurova, Aytekin Yaz, Murat Gumussoy, Orhan G Yigitbasi, Yucel Karaman

**Affiliations:** 1 Department of Otolaryngology and Neck and Head Surgery, The Ministry of Health Izmir Tepecik Training and Research Hospital, 35170, Izmir, Turkey; 2 Department of Anesthesiology, The Ministry of Health Izmir Tepecik Training and Research Hospital, 35170, Izmir, Turkey

## Abstract

**Introduction:**

Anatomic variations of the paranasal sinuses are very common. The paranasal sinus anatomy should be carefully examined prior to performing endoscopic sinus surgery in terms of both existent pathologies and anatomic variations. The anatomy of the paranasal sinuses and its variations have gained importance, along with advances in coronal paranasal sinus computed tomography and extensive use of endoscopic sinus surgery.

**Case presentation:**

A 53-year-old Caucasian woman was admitted to our clinic with complaints of nasal breathing difficulties and headache persisting for a long time. Another concha bullosa was detected in the middle concha bullosa on preoperative paranasal computed tomography examination. It is known that the paranasal sinuses have a number of anatomical variations.

**Conclusion:**

Herein we report a rare case, along with a review of the literature, to emphasize that a concha bullosa inside another concha bullosa should not be ignored.

## Introduction

The importance of the paranasal sinus anatomy and its variations has been emphasized, along with the extensive use of coronal paranasal sinus computed tomography (CT) and endoscopic sinus surgery (ESS). The paranasal anatomy should be exposed in detail prior to ESS to develop treatment strategies during the operation and to prevent possible complications. Attention should be paid to these variations during radiological and endoscopic evaluation of the paranasal sinus anatomy. Concha bullosa is the pneumatization of the middle turbinate and is one of the anatomic variations of the paranasal region [1,2]. Concha bullosa can be either unilateral or bilateral and generally occurs together with a septal deviation to the contralateral side.

Although inferior and superior conchae bullosa have been reported in the literature, this entity is quite rare. The ethmoidal bulla is an anterior ethmoidal sinus cell. Its size, shape and site of drainage may vary among patients. The incidence of middle concha bullosa ranges from 13% to 53% [1,2] and varies according to type. The incidence of bilateral middle concha bullosa has been reported to vary between 45% and 61.5% [1-3]. Herein we present the case of a patient with a large ethmoid bulla extending into a giant middle concha bullosa, which we designate as compound concha bullosa.

## Case presentation

**Figure 1:**
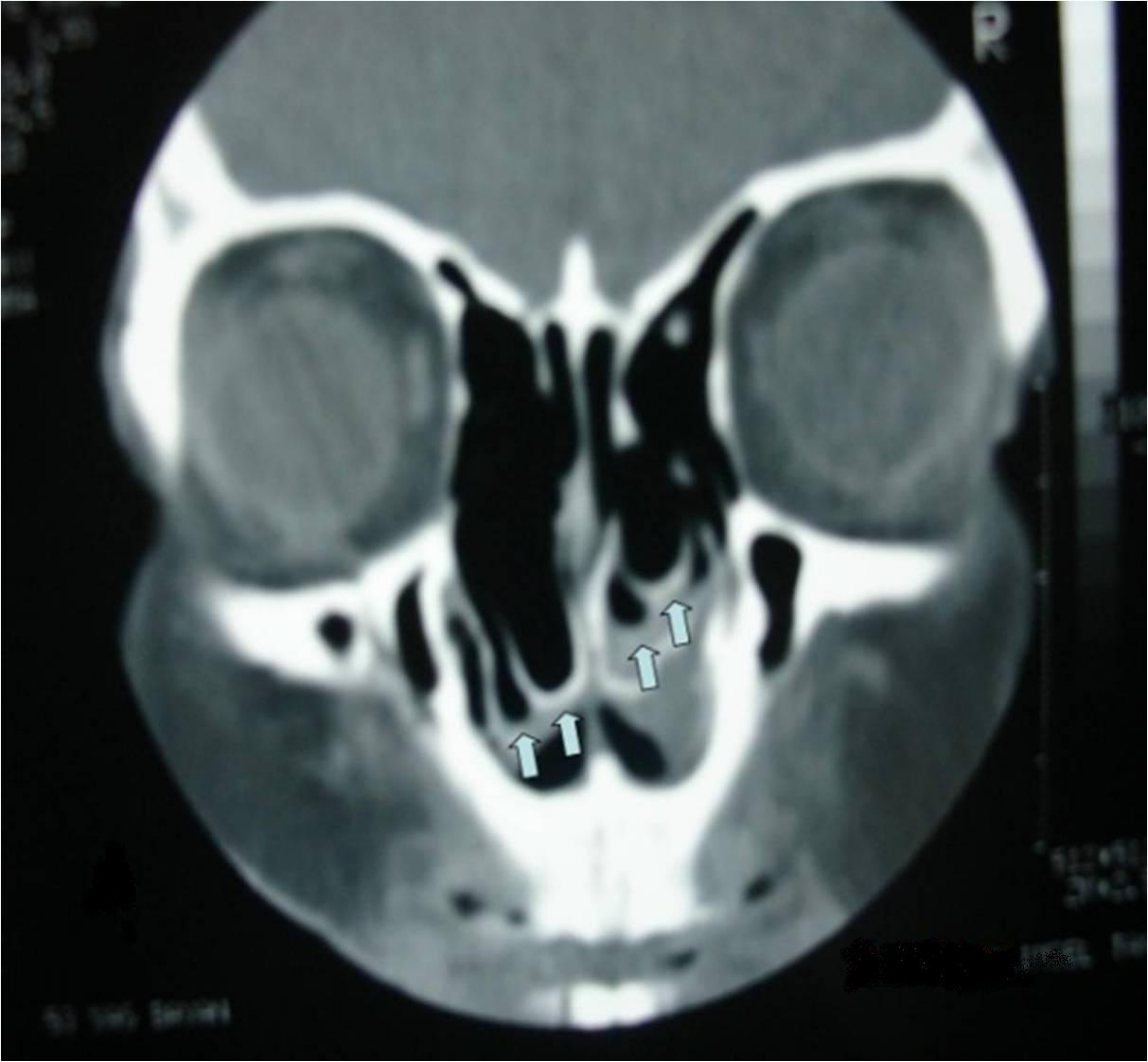
**Coronal paranasal computed tomographic scan showing a concha bullosa inside the bilateral middle concha**. Arrows indicate the inferior aspect of the middle conchae, from which the bilateral conchae bullosa originated.

**Figure 2:**
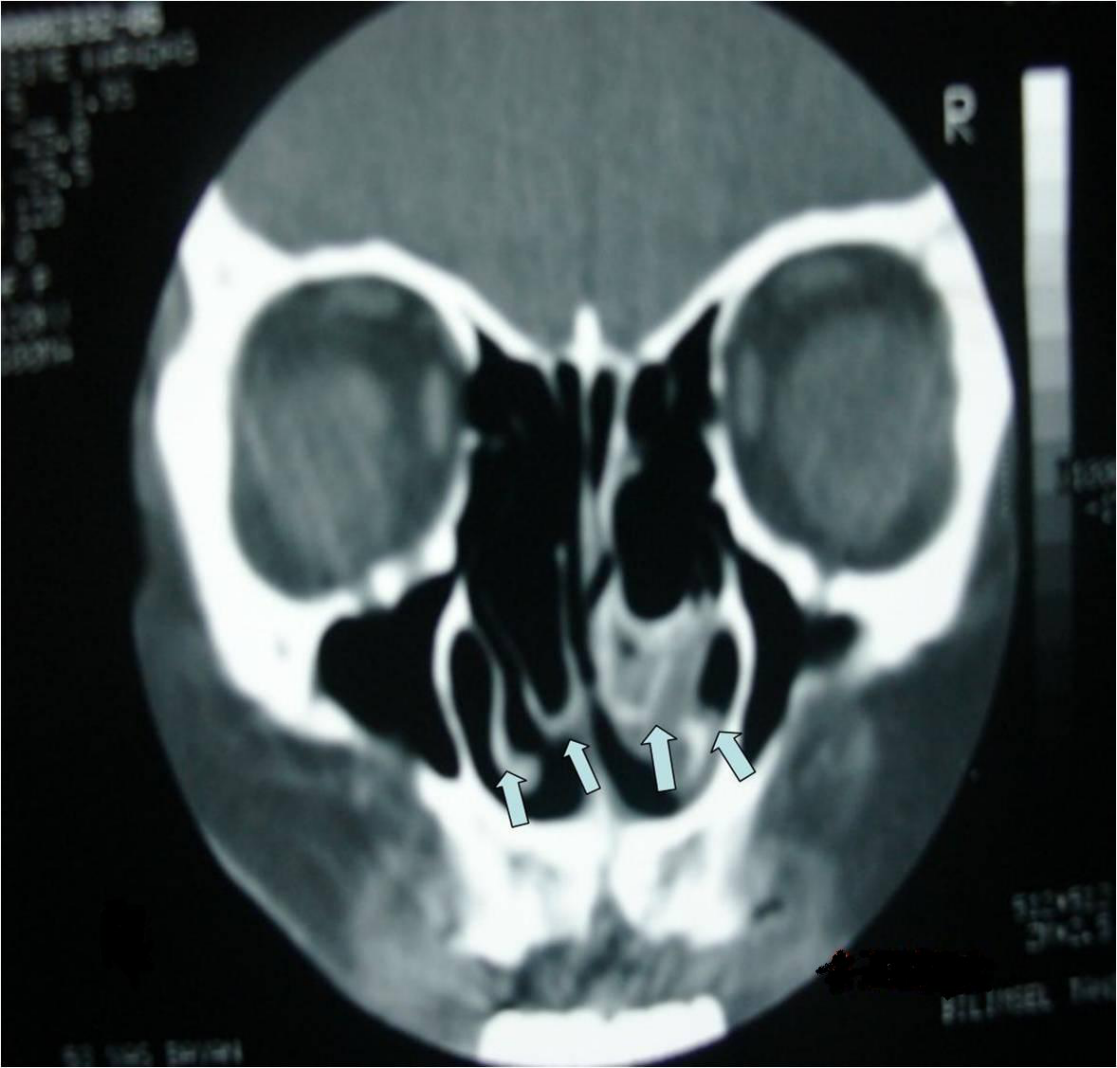
**Coronal paranasal computed tomographic scan from a different angle showing a concha bullosa inside the bilateral middle concha**.

A 53-year-old Caucasian woman was admitted to our clinic with the complaints of nasal breathing difficulties and headache of long duration. She did not have a history of hospital admission or examination for these complaints. Apart from these complaints, she had no other medical problem. An anterior rhinoscopy revealed an anatomy consistent with middle concha bullosa obstructing the bilateral nasal passages, and a septal deviation to the right side was observed. Her coronal paranasal sinus CT scan revealed a giant middle concha bullosa and a large ethmoid bulla extending into the middle concha bullosa on the left side (Figures 1 and 2). A deviation of the septum to the right and a large concha bullosa in the right nasal passage were identified. The patient underwent resection of the concha bullosa and ethmoidal bulla during ESS, and septoplasty was performed. The patient's headache and nasal obstruction complaints were completely relieved within a short time after surgery.

## Discussion

Significant improvements have been made in paranasal sinus surgery, together with advances in endoscopic techniques. However, frequent, miscellaneous anatomic variations in this region increase the risk for possible complications of ESS. Axial and coronal paranasal sinus CT imaging, in addition to endoscopic examination, are of great importance, both for identifying the pathology and for defining regional anatomy and variations prior to the surgery. The ethmoid bone is undoubtedly one of the most complex anatomic structures, and the cells are generally referred to as the anterior and posterior ethmoidal cells according to the site of drainage. However, the anterior and posterior ethmoidal cells also may show a number of variations. The anterior and posterior ethmoidal cells are considered responsible for the pneumatization of the middle concha in approximately 55% and 45% of concha bullosa cases, respectively [1,2].

Bolger *et al. *[2] classified pneumatization of concha bullosa into three groups. They referred to pneumatization localized to the vertical lamella of the middle concha as "lamellar concha bullosa," pneumatization localized to the inferior (or bulbous) pair of the concha as "bulbous concha bullosa," and extensive pneumatization to both the vertical lamella and the bulbous part of the of the concha as "extensive concha bullosa." The degree of pneumatization is directly proportional to the severity of symptoms. Whereas the lamellar and bullous types are usually asymptomatic, extensive bullous concha manifests symptoms [2]. Bolger *et al. *[2] reported the incidences of extensive, lamellar and bulbous concha bullosa to be 15.7%, 46.2% and 31.2%, respectively, but Tonai and Baba [3] reported the incidences of extensive, lamellar and bulbous concha bullosa to be 52%, 28% and 19%, respectively.

Scribano *et al. *[4] reported large ethmoidal bullae in 5.4% of the cases. Concha bullosa is the most common paranasal anatomic variation that causes nasal obstruction and sinusitis. Its prevalence ranges from 8% to 60% [1-4].

On the basis of intraoperative video images, Setliff *et al. *[5] classified 214 ethmoidal bullae into three main categories as simple (47%), compound (26%) and complex (27%) in reference to the association with other ethmoidal cells. They referred to the presence of another cell in the ethmoidal bulla as "complex bulla" [5]. Because of the lack of a distinct posterior wall, Wright and Bolger [6] suggested that the ethmoidal bulla was not a separate cell, but rather a bony lamella.

In this case report, we describe the case of a patient with an extremely rare form of concha bullosa. This form of concha bullosa contains another structure inside (the ethmoidal bulla). The patient had both a large concha bullosa in the right side that obstructed the nasal passage and a large ethmoidal bulla invaginating into the concha bullosa. Although the same variation was seen on the left side, another cell was observed in the giant concha bullosa.

Asymptomatic concha bullosa does not require surgical intervention; however, medical treatment is based on antibiotics, antihistamines and nasal sprays containing topical steroids. Topical decongestants can be given to provide short-term symptomatic relief.

The definitive treatment of concha bullosa is surgical. Although asymptomatic concha bullosa does not require treatment, concha bullosa cases that cause obstruction of the ostiomeatal complex and disease in the paranasal sinuses and those that cause only airway obstruction are treated by performing ESS. Resection of the lateral lamella of the middle concha is an effective procedure and the most commonly used surgical technique [7]. Bhatt [8] advocated a more conservative approach in concha surgery and recommended submucoperiosteal resection.

In our case report, the lateral segments of both bullous conchae were excised endoscopically, and septoplasty was performed. At the patient's control visit 18 months postoperatively, it was observed that her nasal obstruction and headache complaints had been completely relieved. Concha bullosa may not only progress asymptomatically but also present with symptoms such as nasal obstruction, headache and hyposmia by means of completely filling the nasal cavity. Such a large concha bullosa may impair ventilation and drainage of the ostiomeatal complex and thus lead to sinus pathologies.

The relationship of concha bullosa to sinusitis and septum deviation has been the subject of many studies. Aktas *et al. *[9] established a significant relationship between unilateral concha bullosa and the frequency of nasal septal deviation. Uygur *et al. *[10] suggested that septal deviation does not give rise to the formation of concha bullosa, but augments the pneumatization of the middle turbinate, depending on the degree of deviation angle.

Stallman *et al. *[11] also found a strong association between the presence of a concha bullosa and contralateral deviation of the nasal septum, but did not demonstrate a causal relationship, owing to air passage between the concha bullosa and the nasal septum. Moreover, these authors suggested that this association depended on neither the size of concha bullosa nor the degree of septal deviation. Yousem *et al. *[12] reported that the size of concha bullosa, but not its presence, may cause sinusitis. Mucociliary transport of concha bullosa is often directed toward the frontal recess, but rarely to the adjacent air cells or to the hiatus semilunaris. When two mucociliary surfaces contact each other for any reason, mucociliary transport is inhibited in the area of contact. Various studies in the literature have shown that the obstruction of the ostiomeatal complex due to concha bullosa also plays a role as a predisposing factor in sinusitis development [13-15].

## Conclusions

Anatomic variations of the paranasal sinuses are very common. The paranasal sinus anatomy should be examined carefully prior to ESS in terms of existent pathologies and anatomic variations. Nasal diagnostic endoscopy and paranasal CT are of great value in diagnosis. The treatment of such a massive compound concha bullosa, in which an ethmoid bulla has invaginated into a giant concha bullosa, is surgical. Therefore, we think that the present case is a valuable contribution to the literature as a variation that should be considered in the decision to perform ESS.

## Consent

Written informed consent was obtained from the patient for publication of this case report and any accompanying images. A copy of the written consent is available for review by the Editor-in-Chief of this journal.

## Competing interests

The authors declare that they have no competing interests.

## Authors' contributions

IC diagnosed the patient and performed the operation. AY and MG collected data and performed statistical analysis. OGY made a major contribution in writing the manuscript. YK applied the anesthesia. All authors read and approved the final manuscript.
